# COVID-19 Screening Score for Patients without Acute Respiratory Symptoms Undergoing Emergency Medical Procedures in Indonesia

**DOI:** 10.4269/ajtmh.22-0479

**Published:** 2023-05-01

**Authors:** Leonard Nainggolan, Beti Ernawati Dewi, Gerald Abraham Harianja, Yulia Rosa Saharman, Nadira Prajnasari Sanjaya, Robert Sinto, Eric C. M. van Gorp

**Affiliations:** ^1^ Division of Tropical and Infectious Disease, Department of Internal Medicine, Faculty of Medicine Universitas Indonesia – Cipto Mangunkusumo National Hospital, Jakarta, Indonesia;; ^2^ Department of Microbiology, Faculty of Medicine Universitas Indonesia – Cipto Mangunkusumo National Hospital, Jakarta, Indonesia;; ^3^ Department of Internal Medicine, Faculty of Medicine Universitas Indonesia – Cipto Mangunkusumo National Hospital, Jakarta, Indonesia;; ^4^ Department of Viroscience, Erasmus Medical Center, Rotterdam, The Netherlands

## Abstract

To rule out coronavirus disease–2019 (COVID-19) in patients scheduled to undergo emergency medical procedures, SARS-CoV-2 reverse transcription polymerase chain reaction (RT-PCR) must be performed. In developing countries, the use of SARS-CoV-2 RT-PCR has been limited by its unavailability and long processing time. Hence, a quick screening score to predict COVID-19 may help healthcare practitioners determine which patients without acute respiratory symptoms can safely undergo an emergency medical procedure. We conducted a cross-sectional study of adult patients without acute respiratory symptoms who were admitted to the emergency department and underwent an emergency medical procedure within 24 hours after admittance. We collected baseline demographic data, COVID-19 screening variables, and SARS-CoV-2 RT-PCR as the gold standard for COVID-19 diagnosis. Bivariate and multivariate analyses were performed, and a scoring system was developed using statistically significant variables from the multivariate analysis. With data from 357 patients, multivariate backward stepwise logistic regression analysis resulted in two significant COVID-19 predictors: the presence of SARS-CoV-2–IgM antibody (adjusted odds ratio [aOR]: 7.02 [95% CI: 1.49–32.96]) and typical chest x-ray (aOR: 23.21 [95% CI: 10.01–53.78]). A scoring system was developed using these predictors with an area under the receiver operating characteristic curve of 0.71 (95% CI: 0.64–0.78). For a cutoff point of ≥ 2, the scoring system showed 42.5% sensitivity and 97.1% specificity but had poor calibration (Hosmer-Lemeshow test *P* value < 0.001). We believe that the development of this COVID-19 quick screening score may be helpful in a resource-limited clinical setting, but its moderate discrimination and poor calibration hinder its use as a replacement for the SARS-CoV-2 RT-PCR test for COVID-19 screening.

## INTRODUCTION

In March 2020, the WHO declared that coronavirus disease–2019 (COVID-19) had become a global pandemic. In addition to its high transmission rate, diagnosis of COVID-19 has been challenging owing to the variety of clinical presentations, which range from asymptomatic to a critical state with severe respiratory failure and multiple organ failures.^1^^–^^3^ There had been 5–80% COVID-19–infected patients who were asymptomatic yet still as contagious as symptomatic patients.^4^^–^^7^ It is undeniable that the COVID-19 pandemic greatly impacted health services, including access to perform medical procedures normally, especially in developing countries where countermeasures against COVID-19 are still inadequate.^8^^–^^10^

In addition, resources such as diagnostic tools were often scarce, especially early in the COVID-19 pandemic. For example, use of SARS-CoV-2 reverse transcription polymerase chain reaction (RT-PCR), a gold standard for COVID-19 diagnosis, was limited because of long processing times and expensive equipment and testing costs. Access to RT-PCR was especially limited in developing countries, where the test was conducted only in referral hospitals.^11^^,^^12^ Fortunately, some laboratory examinations and imaging studies were more accessible and required shorter turnaround times to get the results, which may have aided clinicians in diagnosing COVID-19 in resource-limited settings.^12^^–^^14^

Several markers from laboratory test results have been recognized and widely used to diagnose COVID-19. Lymphopenia and an increase in the neutrophil-to-lymphocyte ratio (NLR) are two common findings known to have a clinical correlation to COVID-19, and they have played a role in outcome prediction.^2^ Nalbant et al.^15^ showed that the risk of having COVID-19 increased 20.3 times when the NLR was ≥ 2.4. In addition, Yang et al.^16^ suggested that an NLR ≥ 3.3 was a good prognostic factor to predict patients’ deterioration in COVID-19.

C-reactive protein (CRP) is produced by the liver in response to inflammation. In COVID-19 patients, the CRP level may be higher than normal 6–8 hours after the onset of first symptoms and may reach its peak level in 48 hours. In addition, Wang^17^ revealed that CRP levels were positively correlated with lung lesions and reflected the degree of disease severity.

A rapid serologic test for COVID-19 may be an alternative when molecular tests are inaccessible. Spicuzza et al.^18^ showed that a SARS-CoV-2–IgM serology test demonstrated good reliability as point-of-care testing. This might be beneficial in cases where there are discrepancies between clinical/radiological findings and molecular test results. Moreover, patients usually come for treatment in the later phase of the disease; hence, the serologic test may be useful in some cases.

The diagnostic workup for COVID-19 also includes a chest x-ray. It has been suggested that a chest x-ray showing bilateral diffuse reticular-nodular opacity and consolidation with basal and peripheral predomination could indicate a diagnosis of COVID-19 with a sensitivity nearing 68.1%.^13^

Therefore, we aimed to analyze the performance of lymphocyte count, NLR, CRP level, SARS-CoV-2–IgM antibody rapid serology test, and typical chest x-ray in predicting COVID-19 in asymptomatic individuals who underwent emergency medical procedures. We believe all the variables tested are associated with COVID-19, albeit with the exception of respiratory symptoms. Furthermore, we aimed to develop a quick scoring system to diagnose COVID-19 from these parameters.

## MATERIALS AND METHODS

This was a cross-sectional study conducted in Cipto Mangunkusumo National General Hospital, a referral hospital in Jakarta, Indonesia. Inclusion criteria were patients aged 18 years or older who underwent emergency medical procedures in the hospital from April 2020 until March 2021. Patients demonstrating acute respiratory infection symptoms at admission, such as axillary temperature > 37.5°C, cough, and/or dyspnea, were excluded. Ethical clearance with approval number KET-368/UN2.F1/ETIK/PPM.00.02/2021 was obtained from the Health Research Ethics Committee, Faculty of Medicine, Universitas Indonesia, and subjects’ anonymity was ensured.

We collected patient characteristics, including age, gender, body temperature, cough syndrome, dyspnea, and type of medical procedure, as well as COVID-19 screening variables, including lymphocyte count, NLR, CRP levels, SARS-COV-2–IgM serology test, chest x-ray findings (in the form of bilateral diffuse reticular-nodular opacity and consolidation with basal and peripheral predomination), and SARS CoV-2 RT-PCR test. For this study, *medical procedure* was defined as any high-risk aerosol-generating medical procedure, including bronchoscopy, laryngoscopy, tracheal/lung/nasopharynx/oropharyngeal surgery, digestive endoscopy, digestive tract surgery, medical surgery requiring general anesthesia (including caesarean section), ophthalmological procedure, dental procedure, transesophageal echocardiogram, cardiopulmonary resuscitation, cardiopulmonary stress test, and lung function test.^19^^–^^21^ All data were collected within 24 hours before the medical procedure commenced. The data were then analyzed to develop a quick scoring system to predict COVID-19.

Subjects’ characteristics are presented in Table 1. Categorical data are presented as percentage/proportion. The data were then analyzed in the following three steps. First, we performed a bivariate analysis with each variable that became covariate of a positive SARS-CoV-2 RT-PCR result. In this step, we analyzed each covariate in a 2 × 2 table, comparing each covariate in categorical groups with SARS-CoV-2 RT-PCR results (positive/negative). Cutoffs for each covariate were taken from previous studies and the literature.^2^^,^^13^^,^^15^^,^^17^ Second, the odds ratio (OR) was acquired for each covariate, along with the 95% CI and *P*-score. In the next step, any variables with a *P*-score < 0.2 on bivariate analysis were included in the multivariate analysis, which was conducted using logistic regression, and the adjusted OR from each predictor along with the 95% CI and *P*-score were obtained. Variables with a *P* value < 0.05 in the multivariate analysis were included as model variables. Third, we developed a scoring system by dividing the regression coefficient (B) of each model variable by its standard error to obtain the score power of each variable.

**Table 1:** Clinical characteristics of subjects

Characteristic	*n* (%)
Admitting diagnosis	
Acute abdominal pain	19 (5.3)
Musculoskeletal trauma	30 (8.4)
Eye trauma	21 (5.9)
Head injury	18 (5.0)
Burns	10 (2.8)
Hydronephrosis	18 (5.0)
Miscarriage	13 (3.6)
Arrhythmia	5 (1.4)
Acute coronary syndrome	51 (14.3)
Gastrointestinal bleeding	111 (31.1)
Obstructive jaundice	55 (15.4)
Upper respiratory tract bleeding	6 (1.7)
Type of medical procedure	
Digestive surgery	19 (5.3)
Laryngoscopy	76 (21.3)
Cardiovascular intervention	56 (15.7)
Eye treatment	21 (5.9)
Nasopharyngeal surgery	6 (1.7)
Digestive tract endoscopy	166 (46.5)
Caesarean delivery	13 (3.6)

The scoring system was assessed for its discriminatory ability using the receiver operating characteristic (ROC) curve to determine the area under the receiver operating characteristic curve (AUC). An AUC > 0.5 with a *P* value < 0.05 was considered statistically important. The score interval acquired from the scoring system was then categorized to determine the possibility of a COVID-19 diagnosis. The scoring system was then calibrated using the Hosmer-Lemeshow test. All statistical analyses were performed using Statistical Package for the Social Sciences version 23.0 (IBM, Armonk, NY).

## RESULTS

Among 357 patients in this study, 200 (56%) were male and 157 (44%) were female. The median (range) of patients’ age was 49 years (19–88 years). Patients’ clinical characteristics are presented in Table 1.

Table 2 shows bivariate analyses of the COVID-19 screening variables. The variables included lymphocyte count, NLR, CRP level, results of the SARS-CoV-2–IgM antibody rapid serology test, and typical finding on chest x-ray. The SARS-CoV-2 RT-PCR test result was positive in 80 subjects (22.4%), whereas it was negative in the remaining subjects (77.6%). Bivariate analyses were performed with each variable that became covariate to positive SARS-CoV-2 RT-PCR result. From the bivariate analyses, four variables with a *P*-score < 0.2 were obtained: NLR (*P* = 0.002; OR: 2.48 [95% CI: 1.39–4.41]), CRP level (*P* = 0.001; OR: 2.33 [95% CI: 1.40–3.87]), results of the SARS-CoV-2–IgM antibody rapid serology test (*P* < 0.001; OR: 10.14 [95% CI: 2.62–39.22]), and typical chest x-ray (*P* < 0.001; OR: 24.5 [95% CI: 10.82–57.06]). These variables were subsequently included in the multivariate analysis with the logistic regression and backward stepwise method. The multivariate analysis resulted in two statistically significant variables, the presence of SARS-CoV-2–IgM (*P* = 0.013; adjusted OR [aOR]: 7.02 [95% CI: 1.49–32.96]) and typical chest x-ray (*P* < 0.001; aOR: 23.21 [95% CI: 10.01–53.78]), as shown in Table 3.

**Table 2:** Bivariate analyses of COVID-19 screening variables

COVID-19 screening	SARS-CoV-2 RT-PCR, *n* (%)		OR (95% CI)	*P*-score
	Negative	Positive		
Lymphocyte values				
≥ 1,500 cells/mm^3^	157 (77)	47 (23)	–	–
< 1,500 cells/mm^3^	120 (78.4)	33 (21.6)	0.91 (0.55–1.52)	0.742
NLR				
< 2.4	116 (86.6)	18 (13.4)	–	–
≥ 2.4	161 (72.2)	62 (27.8)	2.48 (1.39–4.41)	0.002*
CRP level				
< 10 mg/L	185 (83.3)	37 (16.7)	–	–
≥ 10 mg/L	92 (68.1)	43 (31.9)	2.33 (1.40–3.87)	0.001*
SARS-CoV-2–IgM antibody rapid serology test				
Negative IgM	274 (79.2)	72 (20.8)	–	–
Positive IgM	3 (27.3)	8 (72.7)	10.14 (2.62–39.22)	< 0.001*
Chest x-ray				
Atypical COVID-19	269 (85.4)	46 (14.6)	–	–
Typical COVID-19	8 (19)	34 (81)	24.5 (10.82–57.06)	< 0.001*

COVID-19 = coronavirus disease–2019; CRP = C-reactive protein; NLR = neutrophil-to-lymphocyte ratio; OR = odds ratio; RT-PCR = reverse transcription polymerase chain reaction.

* Statistically significant variable.

**Table 3:** Multivariate analysis of COVID-19 diagnostic predictors

COVID-19 screening	SARS-CoV-2 RT-PCR, *n* (%)		OR (95% CI)	aOR (95% CI)	*P*-score
	Negative	Positive			
NLR					
< 2.4	116 (86.6)	18 (13.4)	–	–	–
≥ 2.4	161 (72.2)	62 (27.8)	2.48 (1.39–4.41)	1.54 (0.81–2.95)	0.182
CRP level					
< 10 mg/L	185 (83.3)	37 (16.7)	–	–	–
≥ 10 mg/L	92 (68.1)	43 (31.9)	2.33 (1.40–3.87)	0.86 (0.42–1.76)	0.681
SARS-CoV-2–IgM antibody serology test					
Negative IgM	274 (79.2)	72 (20.8)	–	–	–
Positive IgM	3 (27.3)	8 (72.7)	10.14 (2.62–39.22)	7.02 (1.49–32.96)	0.013*
Chest x-ray					
Atypical COVID-19	269 (85.4)	46 (14.6)	–	–	–
Typical COVID-19	8 (19)	34 (81)	24.85 (10.82–57.06)	23.21 (10.01–53.78)	< 0.001*

aOR = adjusted odds ratio; COVID-19 = coronavirus disease–2019; CRP = C-reactive protein; NLR = neutrophil-to-lymphocyte ratio; OR = odds ratio; RT-PCR = reverse transcription polymerase chain reaction.

* Statistically significant variable.

After the score of each variable was obtained, a scoring system to predict the diagnosis of COVID-19 was modeled, as shown in Table 4.

**Table 4:** Final analysis of COVID-19 diagnostic predictors

Predictor variable	Regression coefficient (B)	SE	B/SE	Score	Score rounding	*P* value
Positive SARS-CoV-2 IgM	1.950	0.789	2.471	1	1	0.013
Typical chest x-ray finding	3.145	0.429	7.331	2.9	3	< 0.001
Constant	1.827	0.164	–	–	–	< 0.001

COVID-19 = coronavirus disease–2019; SE = standard error.

**Figure 1.**
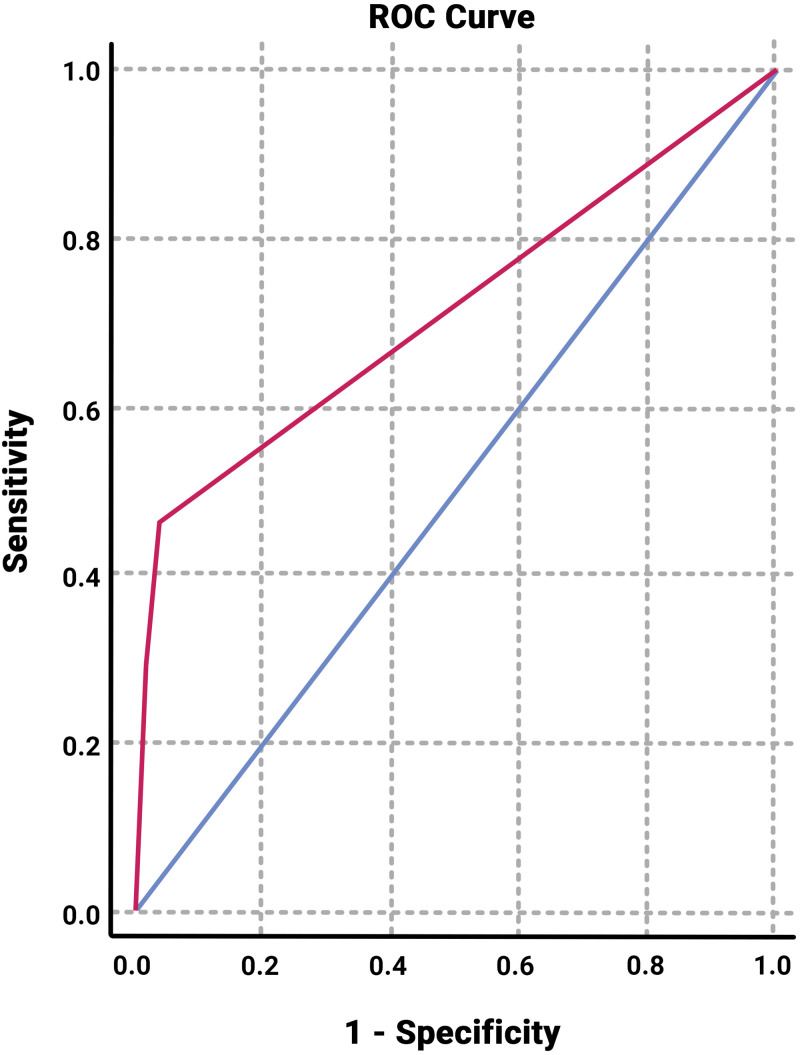
COVID-19 screening scores of asymptomatic patients undergoing a medical procedure, showing an ROC curve with an AUC of 0.71 (95% CI: 0.64–0.78). AUC = area under the receiver operating characteristic curve; COVID-19 = coronavirus disease–2019; ROC = receiver operating characteristic curve.

Figure 1 and Table 5 show the ROC analysis, which assessed the ability of our scoring system to distinguish COVID-19 from non–COVID-19 cases. Cutoffs were obtained on the basis of most optimal sensitivity and specificity. The ROC analysis revealed an AUC of 0.71 (95% CI: 0.64–0.78). However, the Hosmer-Lemeshow calibration test resulted in *P* = 0.000, indicating a significant difference between the number of recruited patients who were predicted to have COVID-19 and those who were not (expected) and the number of recruited patients who positively suffered from COVID-19 and those who did not (observed). Therefore, the scoring system was not well calibrated.

**Table 5:** COVID-19 screening score of asymptomatic patients undergoing a medical procedure

Variable	Category	Score
SARS-CoV-2–IgM antibody serology test	Negative IgM	0
	Positive IgM	1
Chest x-ray	Atypical COVID-19	0
	Typical COVID-19	3
Total score	–	4

COVID-19 = coronavirus disease–2019.

## DISCUSSION

In this study, we developed a quick screening score to predict COVID-19 that can be used in emergency settings where diagnostic tests to determine COVID-19 are limited. Bivariate analysis results suggested four readily available laboratory parameters and imaging studies that may be used to predict COVID-19 in asymptomatic patients—NLR, CRP level, presence of SARS-CoV-2–IgM antibody, and typical findings on chest x-ray—even though most subjects had lymphocyte counts ≥ 1,500 cells/mm^3^ (57.1%), NLR ≥ 2.4 (62.5%), CRP levels < 10 mg/L (62.2%), a negative SARS-CoV-2–IgM antibody serology test result (96.9%), and a normal/atypical chest x-ray (88.2%).

Increased NLR has been demonstrated in symptomatic COVID-19 patients, thus explaining the high NLR level in our subjects. However, other findings may be due to the recruitment of asymptomatic patients in our study. Chen et al.^22^ found that the median (range) lymphocyte count of asymptomatic COVID-19 patients was 1,740 cells/mm^3^ (1,370–2,790 cells/mm^3^), whereas the median lymphocyte count of severe/critical COVID-19 patients was 640 cells/mm^3^ (460–1,030 cells/mm^3^). Yu et al.^23^ revealed that the CRP level differed significantly between asymptomatic and symptomatic COVID-19 patients (0.94 mg/L in the asymptomatic group versus 1.5 mg/L in the symptomatic group). Negative SARS-CoV-2–IgM results were found in the majority of subjects, as previously reported in studies involving asymptomatic patients.^23^^,^^24^ The maturation of immune response usually takes 40 days, and antibody response dynamically depends on the severity of the disease and numerous other factors, explaining falsely negative serology tests during the early stage of COVID-19.^23^ A normal chest x-ray has also been consistently demonstrated in asymptomatic patients.^25^^,^^26^

Further multivariate analyses in the development of our scoring system revealed that only the presence of SARS-CoV-2–IgM antibody and typical findings on a chest x-ray were significantly associated with COVID-19 diagnosis. These two variables were then developed into a scoring system as shown in Table 5, with a moderate AUC performance (AUC: 0.71 with 95% CI: 0.64–0.78). A cutoff point of ≥ 2 had a sensitivity of 42.5% and a specificity of 97.1%. Although our scoring system is highly specific, it was thought that the ideal scoring system for screening purposes should be highly sensitive to provide a ruling in all positive cases. Considering the low sensitivity and poor calibration of our scoring system (Hosmer-Lemeshow test *P* value = 0.000), we suggest that it not be used as a single screening tool to rule out COVID-19 in patients undergoing medical procedures because the low sensitivity of this score may potentially cause a false-negative result in actual COVID-19 patients.

We believe that SARS-CoV-2 RT-PCR remains irreplaceable as a diagnostic tool for COVID-19 despite its limitations and poor accessibility in developing countries. Nowadays, a rapid SARS-CoV-2 antigen test with high sensitivity has emerged as a rapid screening tool in healthcare facilities. According to the literature, the SARS-CoV-2 antigen test may show positive results for patients tested 5 or more days after close contact with SARS-CoV-2 patients; therefore, we do recommend the SARS-CoV-2 antigen test as a primary test for SARS-CoV-2 screening, especially in patients who have acute SARS-CoV-2 but no respiratory symptoms.^27^^,^^28^

To date, this is the first study to investigate the association between lymphocyte count, NLR, CRP levels, rapid serologic testing of SARS-CoV-2–IgM antibodies, and chest x-ray and SARS-CoV-2 RT-PCR in patients without acute respiratory symptoms undergoing medical procedures. However, we realize that data collected from medical records may have limited our study and potentially caused information bias. Another limitation of our study is that the data were conducted during a surge of COVID-19 cases. Theoretically, during a surge, pre-test likelihood may be affected because of the higher prevalence of disease. However, in the clinical setting, where clinicians were assumed to be “blinded” to COVID-19 symptoms because of the inclusion of patients without acute respiratory symptoms, we believe this screening score still has relevance.

In conclusion, we developed this COVID-19 screening score system for use in patients without acute respiratory symptoms undergoing an emergency medical procedure in a medical setting where resources are limited. Many resources, including diagnostic tools such as SARS-CoV-2 PCR tests, are not used as a screening tool because of their unavailability, high cost, and logistical delay. To ensure clinical care and intervention, we created a diagnostic algorithm for excluding SARS-CoV-2 infection. However, its moderate discrimination and poor calibration limit its performance as a replacement for the SARS-CoV-2 RT-PCR test for COVID-19 screening.
